# Dietary Flavonoids and Gastric Cancer Risk in a Korean Population

**DOI:** 10.3390/nu6114961

**Published:** 2014-11-10

**Authors:** Hae Dong Woo, Jeonghee Lee, Il Ju Choi, Chan Gyoo Kim, Jong Yeul Lee, Oran Kwon, Jeongseon Kim

**Affiliations:** 1 Molecular Epidemiology Branch, Division of Cancer Epidemiology and Prevention, Research Institute, National Cancer Center, Gyeonggi-do 410-769, Korea; E-Mails: hdwoo@ncc.re.kr (H.D.W.); jeonghee@ncc.re.kr (J.L.); 2 Center for Gastric Cancer, National Cancer Center Hospital, National Cancer Center, Gyeonggi-do 410-769, Korea; E-Mails: cij1224@ncc.re.kr (I.J.C.); glse@ncc.re.kr (C.G.K.); jylee@ncc.re.kr (J.Y.L.); 3 Department of Nutritional Science and Food Management, Ewha Womans University, Seoul 120-750, Korea; E-Mail: orank@ewha.ac.kr

**Keywords:** gastric cancer, flavonoids, *H. pylori*, smoking, case-control study

## Abstract

Gastric cancer is the most common cancer among men in Korea, and dietary factors are closely associated with gastric cancer risk. We performed a case-control study using 334 cases and 334 matched controls aged 35–75 years. Significant associations were observed in total dietary flavonoids and their subclasses, with the exception of anthocyanidins and isoflavones (OR (95% CI): 0.49 (0.31–0.76), *p* trend = 0.007 for total flavonoids). However, these associations were not significant after further adjustment for fruits and vegetable consumption (OR (95% CI): 0.62 (0.36–1.09), *p* trend = 0.458 for total flavonoids). Total flavonoids and their subclasses, except for isoflavones, were significantly associated with a reduced risk gastric cancer in women (OR (95% CI): 0.33 (0.15–0.73), *p* trend = 0.001 for total flavonoids) but not in men (OR (95% CI): 0.70 (0.39–1.24), *p* trend = 0.393 for total flavonoids). A significant inverse association with gastric cancer risk was observed in flavones, even after additional adjustment for fruits and vegetable consumption in women. No significantly different effects of flavonoids were observed between *H. pylori*-positive and negative subjects. In conclusion, dietary flavonoids were inversely associated with gastric cancer risk, and these protective effects of dietary flavonoids were prominent in women. No clear differences were observed in the subgroup analysis of *H. pylori* and smoking status.

## 1. Introduction

Gastric cancer is a leading cause of cancer mortality in Eastern Asia, Central and Eastern Europe, and Central and South America [1]. Gastric cancer is the most common cancer among men in Korea, and the estimated age-standardized incidence and mortality rates in 2014 were 42.1 and 8.7 per 100,000 persons, respectively [2]. Thus prevention strategies for gastric cancer are a major concern in Korea.

Diet plays an important role in gastric cancer risk. Non-starchy and allium (onion and garlic) vegetables and fruits are most likely associated with reduced gastric cancer risk, while salt and salted foods are most likely associated with an increased risk of this cancer [3]. Leafy vegetables and onions contain high amounts of flavonoids. Onion is a major source of flavonol quercetin, which has a relatively high bioavailability among dietary flavonoid components. The beneficial effects of fruits and vegetables on gastric cancer could be related to flavonoid intake as well as other phytochemicals. We performed a meta-analysis to investigate the effects of dietary flavonoids on gastric cancer risk in a previous study. Total flavonoids and their subclasses except flavonols and quercetin were not significantly associated with a reduced risk of gastric cancer [4]. However, only a small number of papers that reported the effects of dietary flavonoids on gastric cancer risk have been published, and the reported flavonoid subclasses differed across studies. We could not confirm a clear link between dietary flavonoids and gastric cancer risk in the meta-analysis of [5,6,7,8,9,10]. Thus, additional data should be collected to identify the true associations. 

*Helicobacter pylori* (*H. pylori*) and smoking have been classified as causes of cancer of the stomach, according to a monograph from the International Agency for Research on Cancer (IARC) [11,12]. *H. pylori* infection is a major risk factor for gastric cancer, and the growth of *H. pylori* infection was inhibited by flavonoids in an *in vitro* study. Thus, *H. pylori*-induced gastric cancer might be prevented by an appropriate diet. In previous meta-analyses, dietary flavonoids were inversely associated with smoking-related cancer [13] because tobacco contains various carcinogens that can induce free radicals and cause gene mutations in the formation of DNA [14]. The beneficial effects of flavonoids on human health could be from antioxidant activities or regulation of enzymatic pathways. The protective effects of flavonoids on human health cannot be accounted for only by direct antioxidant activities due to low bioavailability. Flavonoids are responsible for the inactivation of phase I enzymes, as well as the activation of phase II enzymes [15]. Thus, dietary flavonoids may play a role in protecting against carcinogens.

Thus, we performed a case-control study to investigate the effects of dietary flavonoids and their subclasses on gastric cancer risk. We included subgroup analyses by *H. pylori* infection and smoking status.

## 2. Experimental Section

### 2.1. Study Population

Cases were defined as histologically confirmed early gastric cancer patients within three months of being recruited at the Center for Gastric Cancer in the National Cancer Center hospital in Korea between April 2011 and May 2014. Patients with diabetes mellitus, other cancer diagnosed within five years, advanced gastric cancer, severe systemic or mental disease, and women who were pregnant and breastfeeding were excluded. Among the 455 subjects who agreed to participate in the study, patients with daily energy intakes of <500 kcal or >5000 kcal (*n* = 3) and those with missing information regarding *H. pylori* (*n* = 5) or smoking status (*n* = 3) were excluded. Controls were recruited from individuals who underwent health-screening examinations at the Center for Cancer Prevention and Detection at the same hospital during the same period. Visitors are beneficiaries of the National Health Insurance program; nearly all Koreans are the beneficiaries of this program. People with cancer, diabetes mellitus, gastric or duodenal ulcer, and previous *H. pylori* treatment were excluded. Among the 899 subjects who agreed to participate in the study, subjects with implausible energy intake (*n* = 6), and missing information in *H. pylori* (*n* = 13) and smoking status (*n* = 4) were excluded. Cases and controls were matched by sex, education (<middle school, high school, and ≥college), and into 10-year age groups. A total of 334 cases and 334 matched controls were finally selected for this study. All participants were provided a written informed consent form, and the study protocol was approved by the Institutional Review Board of the National Cancer Center (IRB protocol number NCCNCS 11-438).

### 2.2. Data Collection and Dietary Assessment

The participants were asked to complete a self-administered questionnaire, which was used to gather information on demographics, lifestyle, and the medical history of the participants. Participants were asked about the average frequency of intake and portion size of specific foods to assess their regular intake during the previous year (*i.e.*, last 12 months) using the validated food frequency questionnaire (FFQ) covering 103 food items [16]. The detailed procedure to design the FFQ is described by Ahn *et al.* [17]. Nine categories of frequency (never or rarely, once a month, two or three times a month, once or twice a week, three or four times a week, five or six times a week, once a day, twice a day, and three times a day) and three portion sizes (small, medium, and large) were specified on the FFQ. After collecting the dietary data, we calculated the individual food intake using CAN-PRO 3.0 (Computer Aided Nutritional Analysis Program, The Korean Nutrition Society, Seoul, Korea). The intake of flavonols, flavones, flavanones, flavan-3-ols, anthocyanidins, and isoflavones were estimated on the basis of the flavonoid database developed by Yang *et al*. [18,19]. Briefly, the flavonoid database was constructed using the United States Department of Agriculture (USDA) flavonoid database, the Korea functional food composition table, and the Japan functional food factor database. Foods that do not have flavonoid contents in those databases were additionally searched for in published papers. Intake of flavonols was the sum of isorhamnetin, kaempferol, myricetin, and quercetin; intake of flavones was the sum of apigenin, and luteolin; intake of flavanones was the sum of eriodictyol, hesperetin, and naringenin; intake of flavan-3-ols was the sum of catechin, epigallocatechin, epicatechin, epicatechin 3-gallate, epigallocatechin 3-gallate, gallocatechin, catechin 3-gallate theaflavin, thearubigins, theaflavin-3,3-digallate, theaflavin-3-gallate, and theaflavin-3′-gallate; intake of anthocyanidins was the sum of cyanidinn, delphinidin, malvidin, pelargonidin, peonidin, and petunidin; and intake of isoflavones was the sum of daidzein, genistein, and glycitein. Total flavonoid intake was the sum of the above flavonoid subclasses. A total of 418 food items from our food consumption data were matched to the flavonoid database. Except for meats, eggs, seafood, and dairy products, which are known to barely contain flavonoid (139 food items), the flavonoid database covered 98% of the consumed food weight of our data. The validity of the FFQ for flavonoid intake has been tested using the three-day dietary record as a gold standard in a total of 202 persons. The crude, energy-adjusted, and de-attenuated energy-adjusted correlation coefficients for total flavonoids were 0.36, 0.25, and 0.32, respectively. *H. pylori* infection was evaluated using a rapid urease test according to the manufacturer’s instructions (Pronto Dry; Medical Instruments Corporation, Solothurn, Switzerland) and by histological evaluation.

### 2.3. Statistical Analysis

Statistical analysis was performed using the SAS version 9.2 statistical package (SAS Institute Inc., Cary, NC, USA). Using a Student *t*-test for continuous variables and a chi-square test for categorical variables, we compared the general characteristics between the case and control subjects. Total flavonoids and their subclasses were adjusted for total energy intake by the regression residual method [20]. Residuals of total flavonoids and flavonoid subclasses were categorized by tertiles, based on the control distribution for the analysis. Odds ratios and 95% confidence intervals for gastric cancer risk were calculated across the tertiles of dietary flavonoids using logistic regression after controlling for known risk factors. The lowest tertile of each dietary flavonoid was used as the reference. Tests for trends across tertiles were performed via the Wald test using continuous variables for each dietary flavonoid (energy-adjusted residual). Model 1 was adjusted for age, total energy intake, *H. pylori* status, occupation, smoking status, alcohol consumption status, BMI, physical activity, and consumption of pickled vegetables and red and processed meat. Model 2 was further adjusted for consumption of fruits and vegetables. Subclass analyses were performed after stratification by *H. pylori* and smoking status.

## 3. Results

The general characteristics of the study participants are reported in Table 1. Cases had a higher total energy intake (*p* < 0.001) and a higher rate of *H. pylori* infection (*p* < 0.001), and they were more likely to be current smokers (*p* < 0.018). No differences were observed between cases and controls with respect to alcohol intake or occupational status.

The ORs and 95% CIs of gastric cancer were analyzed across the tertiles of dietary flavonoids and their subclasses (Table 2). Significant associations were observed in total dietary flavonoids and their subclasses, with the exception of anthocyanidins and isoflavones [OR (95% CI): 0.49 (0.31–0.76), *p* trend = 0.007 for total flavonoids]. However, these associations were not significant after further adjustment for fruits and vegetable consumption [OR (95% CI): 0.62 (0.36–1.09), *p* trend = 0.458 for total flavonoids]. Total flavonoids and their subclasses, except for isoflavones, were significantly associated with a reduced risk gastric cancer in women [OR (95% CI): 0.33 (0.15–0.73), *p* trend = 0.001 for total flavonoids] but not in men [OR (95% CI): 0.70 (0.39–1.24), *p* trend = 0.393 for total flavonoids]. After additional adjustment for fruits and vegetables or vitamin C, none of the associations were significant. In females, gastric cancer risk was significantly associated with flavones after adjustment for fruits and vegetables, and with flavones and flavanones after adjustment for vitamin C (data not shown). Vitamin C and flavonoids were highly correlated each other, and this lead to high multicollinearity.

**Table 1:** General characteristics of the study subjects.

	Controls (*n* = 334)	Cases (*n* = 334)	*P*
Age (y)	51.8 ± 7.6	51.8 ± 9.0	0.996
Male, *n* (%)	208 (62.3)	208 (62.3)	-
Total energy intake (kcal)	1817.7 ± 618.1	2010.2 ± 649.1	<0.001
Physical activity (MET-min/week)	3550.21 ± 4756.69	3429.89 ± 5576.47	0.047
BMI (Kg/m^2^)	24.1 ± 2.9	23.9 ± 3.1	0.210
*H. pylori* infection			<0.001
Negative	159 (47.6)	52 (15.6)	
Positive	175 (52.4)	282 (84.4)	
Smoking status, *n* (%)			0.018
Current	71 (21.3)	103 (30.8)	
Former	104 (31.1)	89 (26.7)	
Never	159 (47.6)	142 (42.5)	
Alcohol intake			0.262
Current	214 (64.1)	208 (62.3)	
Former	19 (5.7)	30 (9.0)	
Never	101 (30.2)	96 (28.7)	
Education, *n* (%)			-
<Middle school	93 (27.8)	93 (27.8)	
High school	146 (43.7)	146 (43.7)	
≥College	95 (28.4)	95 (28.4)	
Occupation, *n* (%)			0.208
Professional	52 (15.6)	62 (18.6)	
Office/service worker	133 (39.8)	107 (32.0)	
Manual worker	63 (18.9)	68 (20.4)	
Other	86 (25.8)	97 (29.0)	
Dietary flavonoids (mg/day)			
Total flavonoids	106.4 ± 82.2	105.2 ± 77.9	0.008 ^†^
Flavonols	22.8 ± 19.5	23.3 ± 21.4	0.042 ^†^
Flavones	1.4 ± 1.1	1.3 ± 1.0	0.021 ^†^
Flavanones	6.4 ± 10.0	5.8 ± 9.5	0.005 ^†^
Flavan-3-ols	22.8 ± 39.2	20.0 ± 35.2	<0.001 ^†^
Anthocyanidins	23.6 ± 25.0	24.9 ± 22.9	0.194 ^†^
Isoflavones	29.4 ± 27.8	29.8 ± 25.0	0.158 ^†^

^†^ Test was performed using each energy-adjusted nutrient residual.

**Table 2:** ORs and 95% CIs of gastric cancer by tertiles of dietary flavonoids.

Tertiles of Energy Adjusted Flavonoid Intake (mg/d) ^†^	Total (*n* = 668)			Men (*n* = 416)			Women (*n* = 252)		
	Controls/Cases	Model 1 OR (95% CI)	Model 2 OR (95% CI)	Controls/Cases	Model 1 OR (95% CI)	Model 2 OR (95% CI)	Controls/Cases	Model 1 OR (95% CI)	Model 2 OR (95% CI)
Total flavonoids (median)									
T1 (52.5)	111/152	1	1	80/99	1	1	31/53	1	1
T2 (73.2)	111/94	0.55 (0.36–0.83)	0.62 (0.39–0.98)	66/61	0.75 (0.44–1.29)	0.80 (0.45–1.43)	45/33	0.49 (0.23–1.05)	0.68 (0.30–1.53)
T3 (152.3)	112/88	0.49 (0.31–0.76)	0.62 (0.36–1.09)	62/48	0.70 (0.39–1.24)	0.80 (0.39–1.63)	50/40	0.33 (0.15–0.73)	0.68 (0.25–1.86)
*p* for trend^‡^		0.007	0.458		0.393	0.977		0.001	0.243
Flavonols (median)									
T1 (10.9)	112/134	1	1	79/91	1	1	33/43	1	1
T2 (14.4)	111/114	0.73 (0.48–1.11)	0.86 (0.55–1.36)	67/74	0.84 (0.49–1.44)	0.89 (0.49–1.61)	44/40	0.55 (0.26–1.20)	0.85 (0.37–1.97)
T3 (30.8)	111/86	0.51 (0.32–0.82)	0.69 (0.39–1.20)	62/43	0.59 (0.32–1.10)	0.65 (0.32–1.35)	49/43	0.51 (0.24–1.10)	1.22 (0.47–3.16)
*p* for trend		0.014	0.543		0.264	0.585		0.017	0.987
Flavones (median)									
T1 (0.6)	111/136	1	1	85/93	1	1	26/43	1	1
T2 (1.0)	112/110	0.69 (0.45–1.06)	0.82 (0.51–1.32)	70/69	0.85 (0.49–1.47)	0.93 (0.50–1.72)	42/41	0.36 (0.15–0.85)	0.42 (0.17–1.03)
T3 (2.1)	111/88	0.51 (0.31–0.82)	0.72 (0.38–1.35)	53/46	0.70 (0.38–1.29)	0.84 (0.37–1.89)	58/42	0.15 (0.06–0.38)	0.22 (0.07–0.67)
*p* for trend		0.004	0.411		0.690	0.385		<.0001	0.010
Flavanones (median)									
T1 (0.7)	111/133	1	1	88/94	1	1	23/39	1	1
T2 (3.5)	112/113	0.85 (0.56–1.28)	0.98 (0.64–1.50)	74/71	0.88 (0.54–1.44)	0.98 (0.58–1.64)	38/42	0.77 (0.33–1.81)	0.90 (0.37–2.15)
T3 (11.0)	111/88	0.66 (0.43–1.02)	0.92 (0.55–1.52)	46/43	0.90 (0.52–1.56)	1.12 (0.58–2.17)	65/45	0.39 (0.18–0.86)	0.64 (0.27–1.52)
*p* for trend		0.032	0.506		0.476	0.987		0.001	0.054
Flavan-3-ols (median)									
T1 (3.2)	111/155	1	1	82/107	1	1	29/48	1	1
T2 (10.8)	111/90	0.61 (0.40–0.92)	0.71 (0.45–1.11)	67/53	0.76 (0.44–1.30)	0.82 (0.45–1.48)	44/37	0.43 (0.20–0.95)	0.62 (0.27–1.44)
T3 (39.7)	112/89	0.58 (0.38–0.88)	0.73 (0.45–1.18)	59/48	0.70 (0.41–1.21)	0.78 (0.41–1.49)	53/41	0.36 (0.17–0.77)	0.65 (0.27–1.57)
*p* for trend		0.001	0.066		0.062	0.175		0.004	0.169
Anthocyanidins (median)									
T1 (9.4)	111/130	1	1	76/79	1	1	35/51	1	1
T2 (14.7)	112/99	0.68 (0.45–1.04)	0.86 (0.54–1.35)	65/65	0.86 (0.50–1.48)	1.01 (0.55–1.83)	47/34	0.53 (0.26–1.10)	0.80 (0.37–1.76)
T3 (35.1)	111/105	0.73 (0.46–1.15)	1.06 (0.62–1.80)	67/64	0.92 (0.51–1.67)	1.16 (0.57–2.34)	44/41	0.58 (0.27–1.25)	1.22 (0.49–3.01)
*p* for trend		0.065	0.976		0.972	0.419		0.005	0.306
Isoflavones (median)									
T1 (11.4)	111/110	1	1	78/67	1	1	33/43	1	1
T2 (19.9)	112/144	1.29 (0.86–1.94)	1.42 (0.94–2.15)	66/98	1.90 (1.12–3.21)	1.99 (1.17–3.38)	46/46	0.64 (0.31–1.34)	0.76 (0.35–1.63)
T3 (43.7)	111/80	0.72 (0.46–1.12)	0.85 (0.54–1.35)	64/43	0.90 (0.52–1.54)	0.98 (0.56–1.73)	47/37	0.51 (0.24–1.08)	0.67 (0.31–1.47)
*p* for trend		0.115	0.400		0.494	0.694		0.059	0.259

^†^ Data were categorized by tertiles of each energy-adjusted dietary flavonoids based on the distribution in controls. ^‡^ Tests for trend across tertiles were performed by Wald test using continuous variables of each dietary flavonoid (energy-adjusted residual). Model 1: adjusted for total energy intake, *H. pylori*, age, sex, education, smoking status, alcohol consumption, BMI, physical activity, and consumption of pickled vegetable and red and processed meat. Model 2: additional adjustment for fruits and vegetable consumption.

**Table 3:** ORs and 95% CIs of gastric cancer by tertiles of dietary flavonoids stratified by *H. pylori* status.

	*H. pylori* Positive				*H. pylori* Negative			
	Men (*n* = 290)	*p* for trend ^†^	Women (*n* = 167)	*p* for trend	Men (*n* = 126)	*p* for trend	Women (*n* = 85)	*p* for trend
Total flavonoids (median)								
Model 1 OR (95% CI)	0.50 (0.25–1.01)	0.193	0.31 (0.11–0.83)	0.005	1.32 (0.41–4.24)	0.996	0.13 (0.02–0.74)	0.022
Model 2 OR (95% CI)	0.57 (0.24–1.33)	0.733	0.78 (0.22–2.84)	0.454	1.37 (0.32–5.76)	0.889	0.30 (0.04–2.17)	0.205
Flavonols (median)								
Model 1 OR (95% CI)	0.55 (0.26–1.17)	0.098	0.48 (0.19–1.21)	0.032	0.61 (0.19–2.01)	0.747	0.34 (0.06–1.81)	0.182
Model 2 OR (95% CI)	0.67 (0.28–1.62)	0.386	1.53 (0.45–5.18)	0.812	0.50 (0.13–1.98)	0.828	0.89 (0.13–6.15)	0.719
Flavones (median)								
Model 1 OR (95% CI)	0.71 (0.34–1.47)	0.769	0.12 (0.04–0.40)	<0.001	0.57 (0.14–2.34)	0.678	0.07 (0.01–0.47)	0.005
Model 2 OR (95% CI)	1.05 (0.40–2.78)	0.201	0.20 (0.05–0.87)	0.025	0.38 (0.07–2.13)	0.527	0.12 (0.01–1.06)	0.056
Flavanones (median)								
Model 1 OR (95% CI)	0.99 (0.51–1.94)	0.784	0.36 (0.14–0.91)	0.003	0.80 (0.25–2.53)	0.242	0.22 (0.03–1.44)	0.010
Model 2 OR (95% CI)	1.45 (0.65–3.24)	0.484	0.62 (0.22–1.72)	0.059	0.68 (0.17–2.68)	0.207	0.52 (0.06–4.38)	0.088
Flavan-3-ols (median)								
Model 1 OR (95% CI)	0.66 (0.35–1.25)	0.132	0.34 (0.14–0.84)	0.007	0.70 (0.22–2.19)	0.113	0.11 (0.02–0.80)	0.050
Model 2 OR (95% CI)	0.80 (0.38–1.72)	0.439	0.64 (0.23–1.82)	0.207	0.58 (0.15–2.25)	0.095	0.28 (0.03–2.55)	0.315
Anthocyanidins (median)								
Model 1 OR (95% CI)	0.85 (0.41–1.75)	0.524	0.85 (0.34–2.15)	0.071	1.05 (0.32–3.45)	0.305	0.04 (0.00–0.42)	0.004
Model 2 OR (95% CI)	1.18 (0.51–2.71)	0.720	2.55 (0.80–8.12)	0.937	1.02 (0.23–4.52)	0.310	0.06 (0.01–0.69)	0.032
Isoflavones (median)								
Model 1 OR (95% CI)	0.77 (0.41–1.44)	0.199	0.48 (0.19–1.18)	0.138	1.12 (0.35–3.55)	0.631	0.50 (0.11–2.17)	0.341
Model 2 OR (95% CI)	0.85 (0.45–1.63)	0.338	0.65 (0.25–1.69)	0.502	1.15 (0.33–4.01)	0.674	0.72 (0.15–3.41)	0.597

^†^ Tests for trend across tertiles were performed by Wald test using continuous variables of each dietary flavonoid (energy-adjusted residual). Model 1: adjusted for total energy intake, age, education, smoking status, alcohol consumption, BMI, physical activity, and consumption of pickled vegetable and red and processed meat. Model 2: additional adjustment for fruits and vegetable consumption.

The associations between dietary flavonoid intake and gastric cancer risk were analyzed after stratification by *H. pylori* (Table 3). No clear differences in the overall effects of dietary flavonoid on gastric cancer risk were found for *H. pylori* infection status (Table 3). *H. pylori*-positive subjects in the highest tertile of total flavonoid and flavan-3-ol consumption were associated with reduced risk in both models, and *H. pylori*-negative subjects in the highest tertile of total flavonoid and flavone consumption were associated with a reduced risk of gastric cancer in the multivariate model. However, the tertile trends for most flavonoids were not significant in either *H. pylori*-positive and negative subjects, except for flavones. Current smokers, former smokers, and never smokers were only compared among male subjects because very few women were ever smokers; the association between flavonoid intake and gastric cancer risk was not significantly different by smoking status (data not shown).

## 4. Discussion

Total dietary flavonoid intake was associated with a reduced risk of stomach cancer, especially among female subjects. However, significant associations were no longer observed except for flavones after adjustment of fruits and vegetable consumption. No clear differences were observed in the subgroup analysis of *H. pylori*.

Our previous meta-analysis, despite the limited number of studies, showed that total flavonoids and their subclasses, except flavonols and quercetin, were not significantly associated with gastric cancer risk [4]. In previous studies, flavonols including the major component quercetin have been shown to be significantly associated with reduced risk of various cancer types. Particularly, due to high bioavailability of quercetin, it has been examined in many cancer studies compared to other sub-classes of flavonoids. In the present study, most flavonoid subclasses were associated with a reduced risk of gastric cancer except for anthocyanidins and isoflavones. The main source of flavonoids could differ according to the food culture of the study subjects. In the present study, the main sources of total flavonoids were green tea, tofu, and black soybeans; flavonols came from onions and radish leaves; flavones were from mandarins and cabbage kimchi; flavanones were from mandarins and orange juice; flavan-3-ols were primarily from green tea; anthocyanidins were from radish root and radish root kimchi; and isoflavones were from tofu and black soybeans. However, the food sources of flavonols, anthocyanidins, and isoflavones were primarily kimchi or fermented soybean paste stew, which have high salt contents. Previous studies [21,22] demonstrated that fermented soy paste and pickled vegetables were associated with increased gastric cancer risk due to high salt intake. Although intake of pickled vegetables was adjusted in the analysis of the models, high intake of salts could negatively affect the beneficial effect of dietary flavonoids. We have included the fruit and vegetable consumption in the model to compare the results with/without adjustment of the variable. Flavonoids and subclasses, with exception of flavones in female, were not significantly associated with gastric cancer after adjustment of fruits and vegetable consumption. Thus, beneficial effects of flavonoids might be closely associated with fruits and vegetable consumption.

In this study, the associations between flavonoids and gastric cancer risk were only significant in the female group. Similarly, the effects of dietary flavonoids on gastric cancer risk were more prominent in female in previous studies. In addition, total dietary flavonoid intake was significantly associated with reduced gastric cancer risk in female but not in male [5], and the strong protective effect of quercetin was also observed in female smokers [6]. In a review of green tea consumption, green tea was not inversely associated with gastric cancer overall, but it was suggested that green tea might be associated with a reduction in gastric cancer risk among women [23]. It was also suggested that men and women have different mechanisms in redox systems [24,25]. Smoking is classified as a cause of gastric cancer [12]; thus, the effect of flavonoids on the smoker group with respect to developing gastric cancer could differ by sex. However, the different effects of flavonoids in women could not be tested because smoking rates were very low among women in this study. The effects of flavonoids on gastric cancer were not significantly different in men by smoking status (data not shown). Growth of *H. pylori* infection was inhibited by flavonoids from licorice extract in an *in vitro* study [26], and *H. pylori* reduces vitamin C bioavailability. Thus, it was expected that dietary flavonoids were differentially associated with gastric cancer risk based on *H. pylori* infection status. However, the associations between flavonoids and gastric cancer risk were not significantly different by *H. pylori* infection status, and these associations did not differ by sex. The sex differences in the effects of flavonoid intake in this study may not be primarily caused by smoking or *H. pylori* infection. The absolute amount of the intake of most flavonoids was higher in women, and energy-adjusted flavonoid intakes were significantly higher in women than men in the present study. In addition, food sources of flavonoids were slightly different between men and women. Flavonoids consumed from pickled vegetables were considerably higher in men, and fruit consumption was lower in men than in women. Thus, different effects of dietary flavonoids on gastric cancer in men and women may be caused by the amount of flavonoid intake and the food sources of flavonoids.

Previous Korean studies investigated the association between dietary flavonoids and disease and found that flavan3-ol intake was inversely related to the risk of metabolic syndrome in women but not in men. However, flavan-3-ol intake was protective against hypertension in non-obese men [19]. The highest tertile of flavan-3-ols exhibited significantly lowered gastric cancer risk compared with the lowest tertile, but linear trends were not observed in either men or women in this study. Serum isoflavones were inversely associated with gastric cancer risk in the Korean population [27] and inhibit cancer cell growth through activation of a signal transduction pathway for apoptosis in an *in vitro* study [28]. However, isoflavone intake was not significantly associated with gastric cancer risk in this study.

The results need to be interpreted with caution due to several limitations. Proanthocyanidins, an abundant group of flavonoids, were not included in total flavonoids database. Bias might be introduced because it was a case-control study. Flavonoid contents in food consumed by the study population might be different from those denoted in the database. The effect of dietary flavonoids cannot be clearly distinguished from that of phytochemicals, such as vitamin C, because they are highly correlated with each other. The bioavailability of each food is different; thus, actual uptake of flavonoids might not be equal. 

## 5. Conclusions 

The protective effects of dietary flavonoids were prominent in women. A clear decreasing trend in gastric cancer risk was observed to be associated with an increase in the dietary intake of total flavonoids, flavones, and anthocyanidins among female subjects. No clear differences were observed in the subgroup analysis of *H. pylori* and smoking status.
